# Unraveling the gut microbiota–SCFAs–cathepsin C pathway in preeclampsia: a novel therapeutic target

**DOI:** 10.3389/fimmu.2025.1700781

**Published:** 2025-11-17

**Authors:** Fan Lu, Houkang Lei, Xiang Xiao, Luzhu Yu

**Affiliations:** 1 Pregnancy Metabolic Disease Laboratory, Affiliated Hospital of GuiZhou Medical University, Guiyang, Guizhou, China; 2 Department of obstetrics and Gynaecology, Zhejiang Provincial People’s Hospital Bijie Hospital, Bijie, Guizhou, China

**Keywords:** preeclampsia, gut microbiota, short-chain fatty acids, cathepsin C, macrophage polarization, immune tolerance

## Abstract

**Introduction:**

Preeclampsia (PE) is a severe obstetric syndrome linked to impaired maternal-fetal immune tolerance, placental insufficiency, and systemic inflammatory activation. Emerging studies suggest that gut microbiota and its metabolites, especially short-chain fatty acids (SCFAs), play a pivotal role in the pathogenesis of PE. However, the precise molecular mechanisms through which SCFAs influence maternal-fetal immune interactions remain poorly understood.

**Methods:**

This study combined clinical data and experimental models to explore the role of SCFAs in regulating cathepsin C expression, a key protease involved in immune modulation, and its impact on immune disturbances in PE. We analyzed the gut microbiota composition and serum SCFA levels in PE patients, and used an L-NAME-induced rat model of PE to assess the effects of SCFA supplementation. Additionally, forced cathepsin C overexpression in rats was performed to establish causality.

**Results:**

Our findings revealed significant gut microbiota alterations in PE patients, with a reduction in SCFA-producing bacteria and an increase in inflammatory microbes. Clinically, SCFA levels were inversely correlated with cathepsin C expression, which was associated with hypertension and proteinuria. In rats, SCFA supplementation significantly reduced cathepsin C levels and alleviated PE symptoms, including hypertension, proteinuria, and fetal growth restriction. Furthermore, overexpression of cathepsin C negated the beneficial effects of SCFAs, exacerbating PE progression. Mechanistically, SCFAs modulated macrophage polarization by inhibiting cathepsin C, promoting the shift to an anti-inflammatory M2 phenotype.

**Discussion:**

This study elucidates the "gut microbiota–SCFAs–cathepsin C–macrophage polarization" pathway as a crucial mechanism in the development of PE. SCFAs promote immune tolerance at the maternal-fetal interface by downregulating cathepsin C and driving M2 macrophage polarization. These findings offer new insights into potential therapeutic strategies for PE, including interventions aimed at modulating the gut microbiota to prevent or mitigate the disease.

## 1 Introduction

Preeclampsia (PE) is a significant pregnancy-related syndrome that involves high blood pressure, proteinuria, and multi-organ dysfunction. With a worldwide incidence ranging from 2% to 8%, it stands as a major contributor to maternal and perinatal health complications and fatalities (1–3). PE is associated not only with severe gestational complications but also with elevated risks to maternal and fetal survival. It has thus become a major factor contributing to global mortality rates among mothers and infants. Core pathological features involve defective restructuring of the spiral arteries in the uterus, dysregulated immune adaptation at the maternal-fetal interface, systemic inflammatory activation (4). While antihypertensive agents and magnesium sulfate can mitigate certain clinical signs, there remains an absence of therapies targeting root causes, underscoring the urgent need to elucidate immune-metabolic pathways in PE to advance treatment strategies.

Accumulating studies implicate gut microbiota dysbiosis in PE pathogenesis via the “gut–placenta axis.” This imbalance is marked notably by a reduced plenty of commensals constructing short-chain fatty acids (SCFAs) and an enrichment of opportunistic pathogens, culminating in declining SCFA (acetate, propionate, butyrate) levels and disrupted immune regulation (5, 6). SCFAs serve as crucial mediators of host-microbiota crosstalk, modulating immune cell activity through two primary mechanisms: engagement of G protein-coupled receptors and reduction of histone deacetylase (HDAC) activity, establishing their role as central regulators of immunological equilibrium (7). Importantly, temporal changes in maternal gut microbial composition and SCFA concentrations correlate strongly with placental development and alterations in immune responses at the maternal-fetal boundary (8). Significant reductions in gut microbial diversity and disturbances in SCFA-producing taxa have been documented in PE patients (9). Moreover, maternal serum SCFA levels are inversely related to disease severity (10), implying that microbiota-derived metabolic alterations may constitute key environmental triggers for PE. Nevertheless, the precise molecular mechanisms through which SCFAs modulate maternal-fetal immune tolerance remain inadequately defined.

Although SCFAs are known to influence immune function, their specific molecular targets at the maternal-fetal interface are not well defined. Notably, cathepsin C—a lysosomal protease found to be elevated in PE placental tissues—may serve as a key intermediary. This cysteine protease plays an essential role in immune activation through its involvement in processing serine proteases, modulating lysosomal proteolytic activity, and regulating intracellular protein turnover (11). Its immunoregulatory importance is further highlighted by observations that cathepsin C-deficient mice display impaired cytotoxic function in NK and T cells (12). Gu Y et al. reported significantly higher Cathepsin C is present in the endothelial cells of blood vessels of pregnant individuals with preeclampsia compared to normotensive controls, noting that such elevated levels promote endothelial kallikrein activation in PE (13). Growing evidence associates cathepsin C with inflammatory conditions, where it facilitates M1 macrophage polarization by activating NF-κB and p38 MAPK signaling, as well as stimulating the NLRP3 inflammasome to amplify inflammatory responses. Our earlier work demonstrated that suppressing cathepsin C alleviates endothelial dysfunction and systemic inflammation in PE (14).Mounting studies indicate that the expression and activity of cathepsins are influenced by the gut microbiota. For instance, Steimle A et al. identified commensal bacteria as major modulators of cathepsin S activity (15), while Bindels LB et al. proved that oral supervision of Lactobacillus reuteri diminishes cathepsin L expression (16). Hence, we postulate that gut microbiota-derived metabolites, including SCFAs, might contribute to PE pathogenesis by regulating cathepsin C.An increased M1/M2 macrophage ratio reflects a key disruption in placental immune tolerance in PE (17). M1 macrophages produce excessive amounts of pro-inflammatory cytokines, including TNF-α and IL-6, aggravating endothelial injury via NF-κB and p38 MAPK activation (18). In contrast, M2 macrophages support immunotolerance and vascular remodeling through IL-4 and IL-10 secretion (19). Although SCFAs are known to promote M2 polarization (20, 21), it remains unclear whether cathepsin C mediates this process.

This study examines clinical correlations between gut microbiota profiles, serum SCFA concentrations, and placental cathepsin C expression in PE patients. Using an L-NAME-induced rat model of preeclampsia, we administered SCFA supplements and performed rescue experiments via forced cathepsin C overexpression to establish causality. Furthermore, in an LPS-stimulated macrophage model, we dissected the molecular mechanism by which SCFAs modulate macrophage polarization through cathepsin C inhibition. Our findings reveal that SCFAs regulate macrophage polarization by suppressing cathepsin C, providing new theoretical support for early risk detection and targeted therapy in PE.

## 2 Materials and methods

### 2.1 Clinical sample collection

The study protocol obtained approval from the Ethics Committee of the Affiliated Hospital of Guizhou Medical University (Approval No.: 2023-604). A total of twenty pregnant women who have been identified as having preeclampsia (PE group) and twenty healthy pregnant women serving as normal controls (NC group) were enrolled, each participant provided written consent after being informed. Peripheral venous blood (5 mL, collected in EDTA-coated tubes) and freshly voided fecal specimens (stored in sterile containers) were obtained from each subject within 24 hours prior to delivery. Serum was divided using a centrifuge at 3000 rpm for 10 minutes and preserved at –80°C. Stool specimens were rapidly snap-frozen in liquid nitrogen, then relocated to –80 °C ultra-low temperature freezers for extended storage. Inclusion criteria comprised singleton pregnancies without additional obstetric complications. Exclusion criteria included current infectious or metabolic diseases, as well as recent antibiotic usage.

### 2.2 16S rRNA sequencing

Collected fecal samples were promptly transported and maintained at –80 °C until processing. Total microbial genomic DNA was extracted and subjected to 16S rDNA sequencing. Following PCR amplification, amplicons were purified, and sequencing libraries were constructed and quality-checked. Sequencing was carried out on the Illumina NovaSeq system. The obtained reads were grouped into operational taxonomic units (OTUs) with 97% sequence identity and subsequently annotated using the SILVA reference database. Differences in microbial community composition, α- and β-diversity indices, and the abundance of short-chain fatty acid–producing taxa were compared between PE and NC cohorts.

### 2.3 Cell culture

RAW264.7, a mouse macrophage cell line, was purchased from AnWei-sci (Cat# AW-CH0273). Cells were propagated in RPMI-1640 medium (Gibco, USA) supplemented with 10% fetal bovine serum (FBS, Gibco, USA) and 1% penicillin–streptomycin, under conditions of 37 °C and 5% CO_2_ in a humidified incubator. Authentication of all lines was confirmed through short tandem repeat (STR) profiling.

### 2.4 Cell transfection

A lentiviral vector system (pLVX-Puro, General Biol, China) was used to generate a RAW264.7 cell line stably overexpressing murine Cathepsin C. Cells were introduced into culture plates and co-transduced with recombinant lentivirus carrying the Cathepsin C sequence and Polybrene with a concluding concentration of 6–8 μg/mL. After 24–48 hours, transduced cells were chosen using a full medium with 2 μg/mL puromycin over a period of 14 days. Once all cells in the control group had died, a stable polyclonal population was established. Overexpression efficiency was confirmed through quantitative real-time PCR (qRT-PCR) at the levels of mRNA and protein. Cells transduced with empty vector (oe-NC group) served as the control.

### 2.5 Rat model of preeclampsia

**Figure 1:**
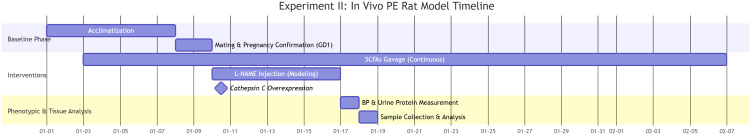
Experimental timeline and design of the SCFA and cathepsin C intervention in the L-NAME-induced rat model of preeclampsia.

Approval for all animal procedures was granted by the Animal Ethics Committee of Guizhou Medical University (Approval No.: 2304792). After a 7-day acclimatization period under specific pathogen-free (SPF) conditions, 8–10-week-old Wistar rats were mated. The presence of sperm in vaginal smears defined gestational day 1. Starting on gestational day 7, pregnant rats received daily subcutaneous injections of the nitric oxide synthase inhibitor L-NAME (250 mg/kg/day for 7 days) to induce preeclampsia. The L-NAME model was selected for its well-established ability to reliably recapitulate core PE phenotypes, including hypertension, proteinuria, and fetal growth restriction, primarily through induction of endothelial dysfunction. Concurrently, the SCFA-treated groups received a daily oral gavage of an SCFA mixture (0.1 mL per 10 g body weight) containing 67.5 mM sodium acetate, 25.9 mM sodium propionate, and 40 mM sodium butyrate. This dosage and formulation, based on previous studies and our pilot experiments, were designed to achieve a physiologically relevant increase in circulating SCFA levels, mirroring the effects of a high-fiber diet and enhancing the translational potential of our findings. The animals were organized into five distinct groups(n=9 pregnant rats per group):NC group: no treatment; PE group: L-NAME only; SCFA group: L-NAME + oral gavage of SCFA mixture (Administer daily for 35 days, 0.1 mL per 10 g body weight, with a composition of 67.5 mM sodium acetate, 25.9 mM sodium propionate, and 40 mM sodium butyrate); Vector group: L-NAME + empty lentivirus; Intervention group: L-NAME + SCFA mixture + Cathepsin C overexpression lentivirus. After the experimental period, fetal and placental weights and lengths were recorded. The schematic overview of the experimental timeline design is presented in **Figure 1**.

### 2.6 Urinary protein and blood pressure measurements

Urinary protein levels were assessed using 24-hour urine collections. Total protein was precipitated with sulfosalicylic acid and quantified using an automatic biochemical analyzer (OLYMPUS-AU400).Blood pressure was measured non-invasively with a CODA tail-cuff system. Rats were acclimated in a temperature-controlled restraint platform, and three measurements of SBP and DBP were taken at five-minute intervals following stabilization, the average value was recorded for analysis.

### 2.7 Hematoxylin and eosin staining

Placental tissue paraffin sections underwent dewaxing in xylene solutions (I and II) for 10 minutes per step, followed by rehydration using a graded ethanol series (100% to 75% ethanol). Subsequently, the sections underwent hematoxylin staining for 5 min, followed by rinsing with running water and differentiation using 1% HCl-alcohol for 30s (stopping when the nuclear staining was clear examined with a microscope), then rinsed with tap water and blued for 10 minutes. The sections underwent eosin staining for 2 minutes, dehydrated through a gradient of ethanol (75% to 100%) for 2 min per step, cleared using xylene for 5 minutes and then mounted in neutral gum.

### 2.8 ELISA

Following a 10-minute centrifugation at 1000×g, the supernatants from the samples were harvested. The cell lysates were treated with RIPA buffer and then centrifuged (Subject to 12000 g for a period of 10 minutes) to collect the supernatants, divide them into portions, and store at -80 °C. The capture antibody (1-10 μg/mL) was immobilized onto a 96-well plate and allowed to sit overnight at 4°C. Following a 1-hour blocking period with a blocking solution containing 5% BSA, the diluted samples (1:10 dilution) or the standard dilution series were added sequentially and kept at 37 °C for an hour. Subsequently, the plate underwent three wash cycles. Then, the enzyme-labeled secondary antibody (HRP-conjugated, 1:1000) introduced and allowed to incubate for 60 minutes. The plate was then subjected to five washing steps. Following this, the TMB substrate was applied for color development, which proceeded for 10 minutes under light-protected conditions. The reaction was terminated using 2M H_2_SO_4_, after which the absorbance at 450 nm was immediately measured. Blank wells, negative and positive controls were set up in the experiment. The standard curve was calculated by 4-parameter Logistic fitting to determine the sample concentration. Each sample was measured in duplicate (technical replicates), and all experiments were repeated independently at least three times (biological replicates, n=3).

### 2.9 Flow cytometry

After collecting RAW264.7 cells, they underwent two washes with pre-cooled PBS to eliminate cellular debris. Fc receptor blocking agent (Anti-mouse CD16/32 antibody, 1 μg/test) was introduced and incubated on ice for 10 minutes to reduce non-specific binding. Then, surface marker antibodies APC-CD86 (1 μg/test), PerCP-Cy5.5-CD11b (0.25 μg/test), and F4/80 (AF488 labeled, 1 μg/test) were added in sequence and maintained in a light-shielded setting at 4 °C for 30 minutes. Following PBS washing, the cells were immediately suspended in flow cytometry staining buffer. The co-expression ratio of CD86 (an M1 macrophage marker) and CD163 (an M2 macrophage marker) in CD11b+F4/80+ cell populations were assessed using flow cytometry, with the percentage of double-positive cells determined through analysis of multiple fluorescent channels. The staining and analysis were performed on cells from three independent cell cultures (biological replicates, n=3). For each experiment, at least 10,000 events in the target lymphocyte or macrophage gate were acquired.

### 2.10 Immunofluorescence

Placental tissues were fixed in 4% paraformaldehyde and embedded in paraffin. Sections (4–5 μm) were deparaffinized and rehydrated. Antigen retrieval was performed using EDTA buffer (pH 9.0) under high-pressure heating (121°C, 3 min). After cooling and PBS washing, sections were blocked with 10% normal goat serum for 1 h at room temperature, followed by incubation with primary antibody overnight at 4°C. To confirm the specificity of immunostaining, negative control sections were processed in an identical manner but with the primary antibody omitted; no significant nonspecific staining was observed in these controls. After PBS washes, sections were incubated with Alexa Fluor 488- or 594-conjugated secondary antibodies (1:500) for 1 h in the dark. Nuclei were counterstained with DAPI, and slides were mounted with antifade medium. Imaging was performed using a fluorescence microscope. Representative images from at least three different placental tissue samples per group (biological replicates, n=3) are shown. For each sample, three random fields of view were captured and analyzed.

### 2.11 Western blot analysis

Tissues were rinsed with ice-cold PBS to remove residual blood, then rapidly frozen in liquid nitrogen and ground into powder. RIPA lysis buffer containing protease and phosphatase inhibitors (PMSF plus inhibitor cocktail) was added at a ratio of 1:10 (tissue to buffer), followed by homogenization on ice for 30 minutes. Lysates were centrifuged at 12,000 rpm for 15 min at 4°C, and protein levels were determined using the BCA assay. After mixing with loading buffer, the samples were boiled to induce protein denaturation. Electrophoresis was performed by SDS-PAGE, with gel percentage adjusted according to protein size (e.g., 10% gel for β-actin; 80 V stacking and 120 V separating). Proteins were subsequently transferred to PVDF membranes using wet transfer at 180 mA for 90 min. Membranes were blocked with 5% skim milk at room temperature for 1 h before overnight incubation at 4 °C with primary antibodies. After three washes in TBST, HRP-conjugated secondary antibodies (1:5000) were applied for 1 h at room temperature. Signal detection was achieved by ECL chemiluminescence, and band intensities were quantified with ImageJ software. Protein extracts were prepared from three independent tissue samples or cell cultures per group (biological replicates, n=3). The blot shown is representative of these three independent experiments.

### 2.12 Antibodies

All primary and secondary antibodies used for flow cytometry, immunofluorescence, and Western blot analysis are listed in **Supplementary Table 1**, including supplier, catalog number and dilution used.

### 2.13 Statistical analysis

Statistical analyses were performed using SPSS 26.0 and GraphPad Prism 9.0. Continuous data are presented as mean ± standard deviation (SD). After verifying data normality with the Shapiro-Wilk test, specific statistical tests were chosen based on the experimental design and data characteristics. The unpaired Student’s t-test was applied for comparing two independent groups with normal distributions. Comparisons across multiple groups in animal or cell studies utilized one-way analysis of variance (ANOVA) followed by Tukey’s *post-hoc* test. The longitudinal nature of the blood pressure data, involving repeated measurements from the same subjects over time, necessitated the use of repeated-measures ANOVA to properly account for within-subject correlations; Bonferroni correction was applied for multiple comparisons. For data that violated the assumption of normality or were expressed as percentages, the Mann-Whitney U test was employed, with data transformations used when necessary. Associations among SCFAs, cathepsin C, and clinical indices were assessed using Spearman’s rank correlation.

The threshold for statistical significance was set at a P value less than 0.05, with asterisks denoting levels of significance. To address the reviewer’s suggestion and provide a more complete interpretation of our results, we have reported effect sizes alongside P values. Cohen’s d for group comparisons and Pearson’s r for correlations, each accompanied by their 95% confidence intervals, are included in the Results section.

The sample size for the clinical cohort was primarily determined by feasibility and patient availability. For the animal experiments, the group size was selected in accordance with established standards in the field and was supported by our pilot data, which indicated this number would be sufficient to detect significant differences in key parameters. Although an *a priori* power calculation was not conducted, *post-hoc* power analyses for primary outcomes confirmed a statistical power exceeding 80%. The robustness of the findings is further supported by the large effect sizes observed. We acknowledge that the current study was not powered to enable a comprehensive multivariate analysis that controls for all potential confounders, an important objective for future validation in larger cohorts.

## 3 Results

### 3.1 Significant alterations in gut microbial composition in PE

**Figure 2:**
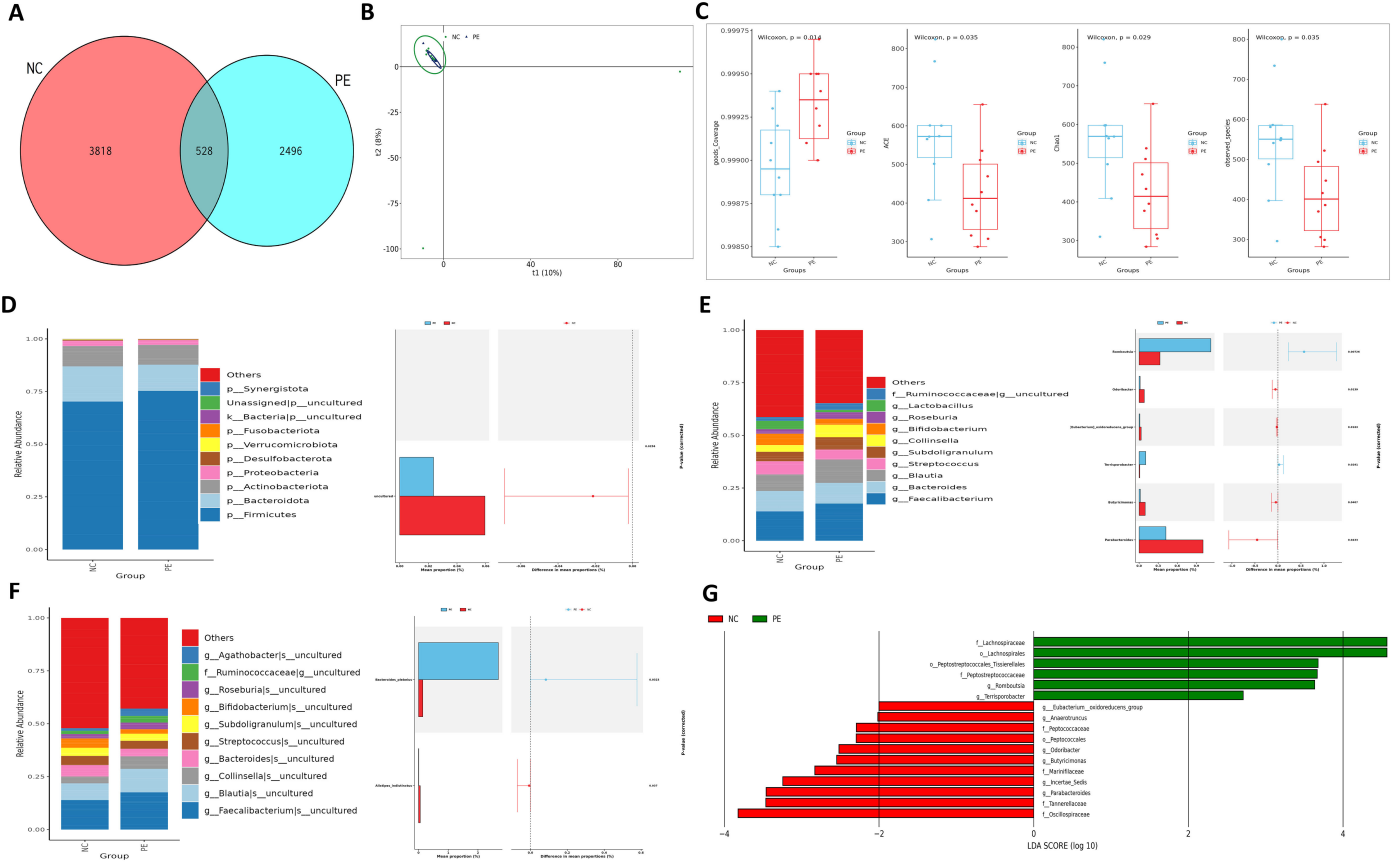
Intestinal ecological imbalance in patients with PE. **(A)** Venn diagram showing the number of shared and unique Amplicon Sequence Variants (ASVs) between groups. **(B)** Principal Component Analysis (PCA) of β-diversity illustrating distinct clustering of microbial communities in PE and NC groups. **(C)** Box plots comparing α-diversity indices (ACE, Chao1, Coverage, Observed species) between groups, indicating reduced microbial richness and diversity in PE. **(D)** Relative abundance of bacterial phyla. Note the decreased Firmicutes/Bacteroidota ratio and increased abundance of Proteobacteria in the PE group. **(E, F)** Heatmaps displaying the relative abundance of the top 10 significantly different bacterial genera **(E)** and species **(F)** between groups. **(G)** Cladogram from LEfSe analysis identifying the most differentially abundant bacterial taxa, with the NC group enriched in SCFA-producing families like Lachnospiraceae. Data are presented as mean ± SD (n=20). Statistical significance was determined by Student’s t-test or Mann-Whitney U test.

To investigate the role of gut microbiota in PE, we first performed 16S rRNA sequencing on fecal samples from PE patients and normotensive controls (NC). Our analysis revealed substantial alterations in the gut microbial community structure in the PE group (**Figure 2A**). A marked decline in microbial α-diversity was observed in the PE group compared to the NC group (**Figure 2C**). β-Diversity analysis further indicated a clear separation between the two groups (**Figure 2B**), reflecting considerable disruption of ecological equilibrium in the gut microbiome of individuals with PE. In terms of phylum, a diminishment in Firmicutes and an expansion of Proteobacteria were detected. This shift showed a strong correlation with elevated blood pressure and impaired placental function. Genus-level examination identified a pronounced decrease in key SCFA-producing taxa, including butyrate-producing Blautia, propionate-producing Collinsella (50% reduction), and Akkermansia muciniphila. Quantification at the species level confirmed a significant decline in beneficial organisms such as Alistipes indistinctus. LEfSe analysis highlighted an enrichment of pro-inflammatory genera including Parabacteroides and Odoribacter in the PE group. Alternatively, the NC group demonstrated a higher prevalence of SCFA-producing genera, including Romboutsia and multiple Lachnospiraceae family members (**Figures 2D–F**).

**Figure 3:**
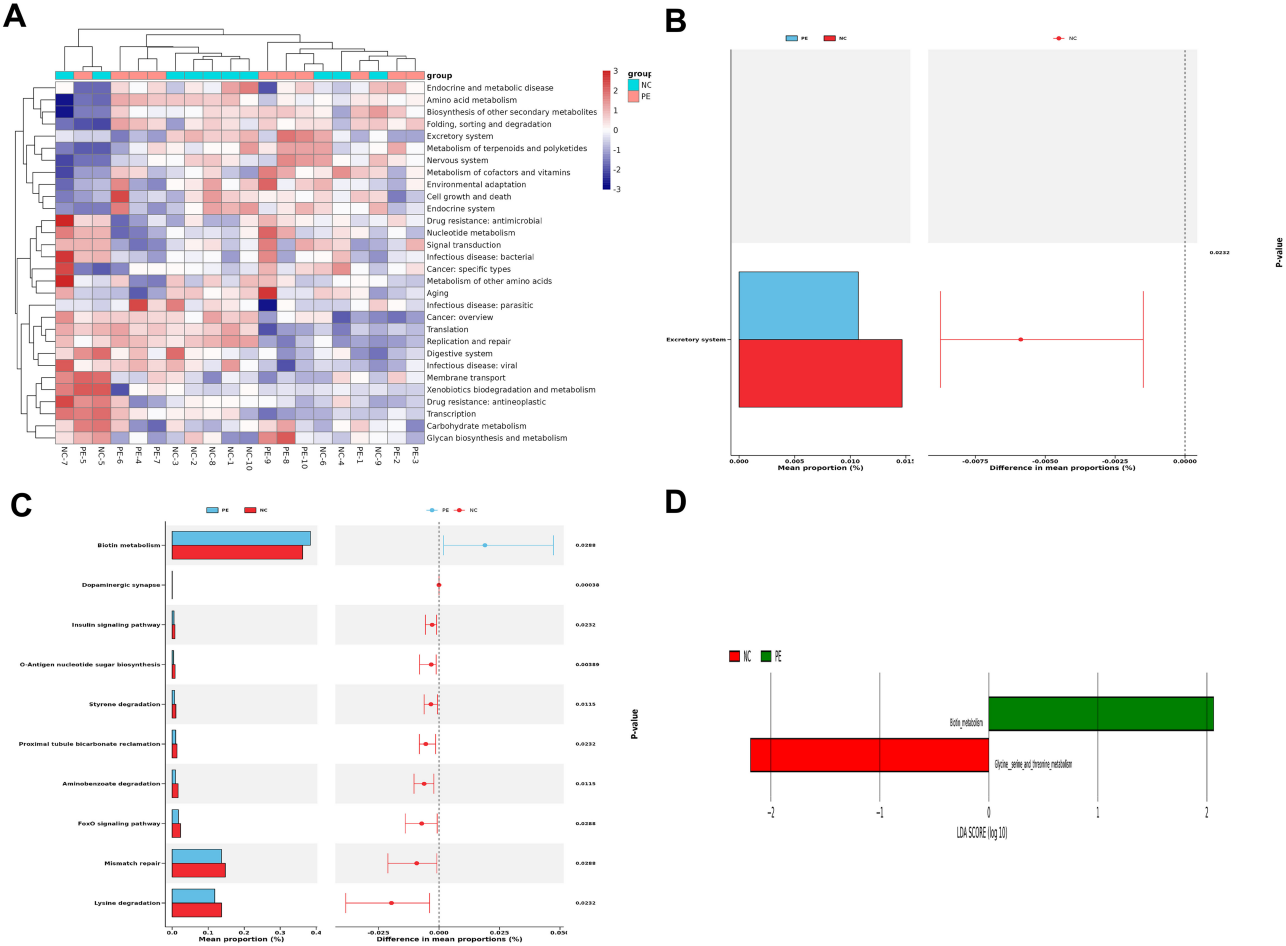
Distinct Functional Alterations in the PE Gut Microbiome. Functional profiling was performed using PICRUSt2 based on KEGG orthology. **(A)** Heatmaps of functional clustering at Level 2, demonstrating distinct metabolic profile separation between groups. **(B,C)** STAMP differential abundance analysis of Level 2 **(B)** and Level 3 **(C)**pathways. Bar plots represent the mean proportion of each pathway, and whisker plots indicate the 95% confidence intervals. **(D)** LEfSe analysis highlighting the most differentially enriched KEGG pathways at Level 3. Pathways related to carbohydrate metabolism and SCFA biosynthesis (Glycine, serine and threonine metabolism) were significantly depleted in the PE group. Group A (NC), n=20; Group B (PE), n=20.

Functional profiling demonstrated dysregulation of excretory pathways and significant suppression of metabolic routes involved in SCFA biosynthesis in the PE cohort. Pathways related to glycine and serine metabolism—precursors of butyrate—were notably enriched in NC subjects, whereas biotin metabolism was aberrantly elevated in PE patients (**Figure 3**; **Supplementary Figure S8**). These compositional and functional disruptions suggest that reduced Firmicutes and elevated Proteobacteria contribute to microbial imbalance. Diminished SCFA-producing capacity and metabolic pathway dysregulation may underlie PE pathogenesis via impaired immune modulation and placental development.

### 3.2 Decline in SCFA-producing taxa is linked to clinical manifestations of PE

Building on the overall dysbiosis, we specifically analyzed the abundance of SCFA-producing taxa using our 16S rRNA sequencing data. Key bacterial genera instrumental in SCFA synthesis were markedly decreased in the gut microbiota of individuals with PE, as evidenced by 16S rRNA sequencing. Butyrate-producing Blautia and propionate-producing Collinsella were less abundant, while acetate-producing Romboutsia was more prevalent in the NC group (**Figure 2G**). KEGG annotation revealed notable downregulation in the glycine/serine metabolic pathway—essential for butyrate production—and reduced activity in lysine degradation, which is linked to propionate metabolism, in the PE group (**Figure 3**; **Supplementary Figure S8**). These findings reflect impaired microbial capacity for SCFA synthesis in PE. Given the recognized role of SCFAs in suppressing HDAC activity and modulating immune responses, the depletion of SCFA-producing bacteria and subsequent SCFA deficiency may contribute to hypertension and placental dysfunction in PE by attenuating protective effects on vascular endothelium and trophoblastic function.

### 3.3 Systemic inflammation and elevated placental cathepsin C in PE patients

**Figure 4:**
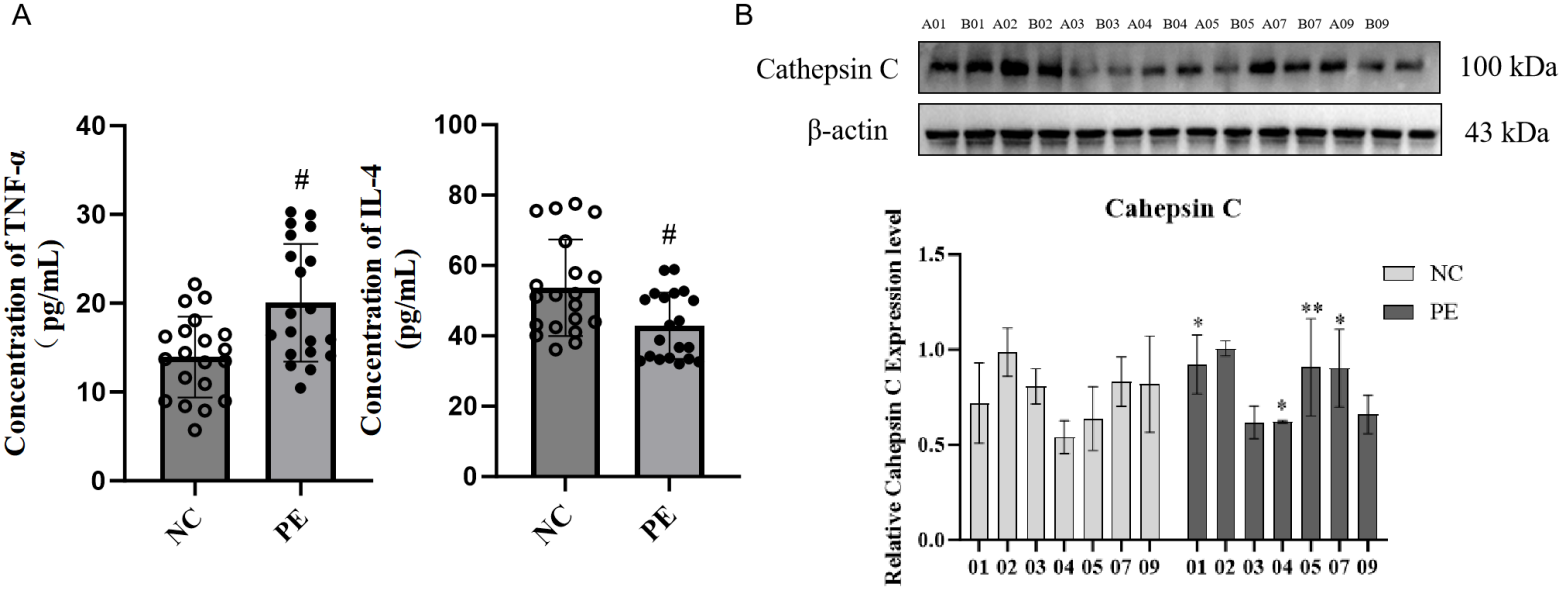
Systemic inflammation and elevated placental Cathepsin C in PE patients. **(A)** Serum concentrations of the pro-inflammatory cytokine TNF-α and the anti-inflammatory cytokine IL-4, as determined by ELISA. PE patients exhibited a pro-inflammatory cytokine shift. **(B)** Representative western blot (left) and quantitative analysis (right) of Cathepsin C protein expression in placental tissues. GAPDH was used as a loading control. Data are presented as mean ± SD(n=20). Statistical significance was determined by Student’s t-test; ^*^P < 0.05, ^**^P < 0.01, ^***^P < 0.001 vs. Control group. ^#^P < 0.05 vs. LPS group.

To assess the systemic and placental inflammatory status, we measured cytokine levels in serum and cathepsin C expression in placental tissues from our clinical cohort. Analysis of peripheral blood from PE patients revealed significantly elevated levels of the pro-inflammatory cytokine TNF-α and decreased amounts of the anti-inflammatory cytokine IL-4 (both P < 0.05; **Figure 4A**), reflecting a systemic shift toward a pro-inflammatory state. Further assessment of placental tissues indicated pronounced upregulation of cathepsin C expression compared to the normal pregnancy group (P < 0.05; **Figure 4B**). As a canonical pro-inflammatory mediator, TNF-α may enhance trophoblast apoptosis and foster a pro-inflammatory microenvironment by increasing cathepsin C expression. In turn, cathepsin C may contribute to the degradation of extracellular matrix components, potentially worsening placental barrier integrity. This reciprocal interaction offers new molecular perspective into the mechanisms driving defective placental implantation and maternal vascular endothelial injury in PE.

### 3.4 SCFA supplementation alleviates PE symptoms in rats via cathepsin C downregulation

To assess the causal role of gut microbiota-derived SCFAs and cathepsin C in PE, we conducted multi-arm interventions in an L-NAME-induced rat model. Pregnant rats were divided into five groups: the normal control (NC) group, the PE model group (PE), the PE model treated with an SCFA mixture (PESCFAs27), the PE model transfected with an empty vector (PE+oe-NC), and the PE model treated with SCFAs while overexpressing cathepsin C (PESCFAs27+oe-Cathepsin C).

**Figure 5:**
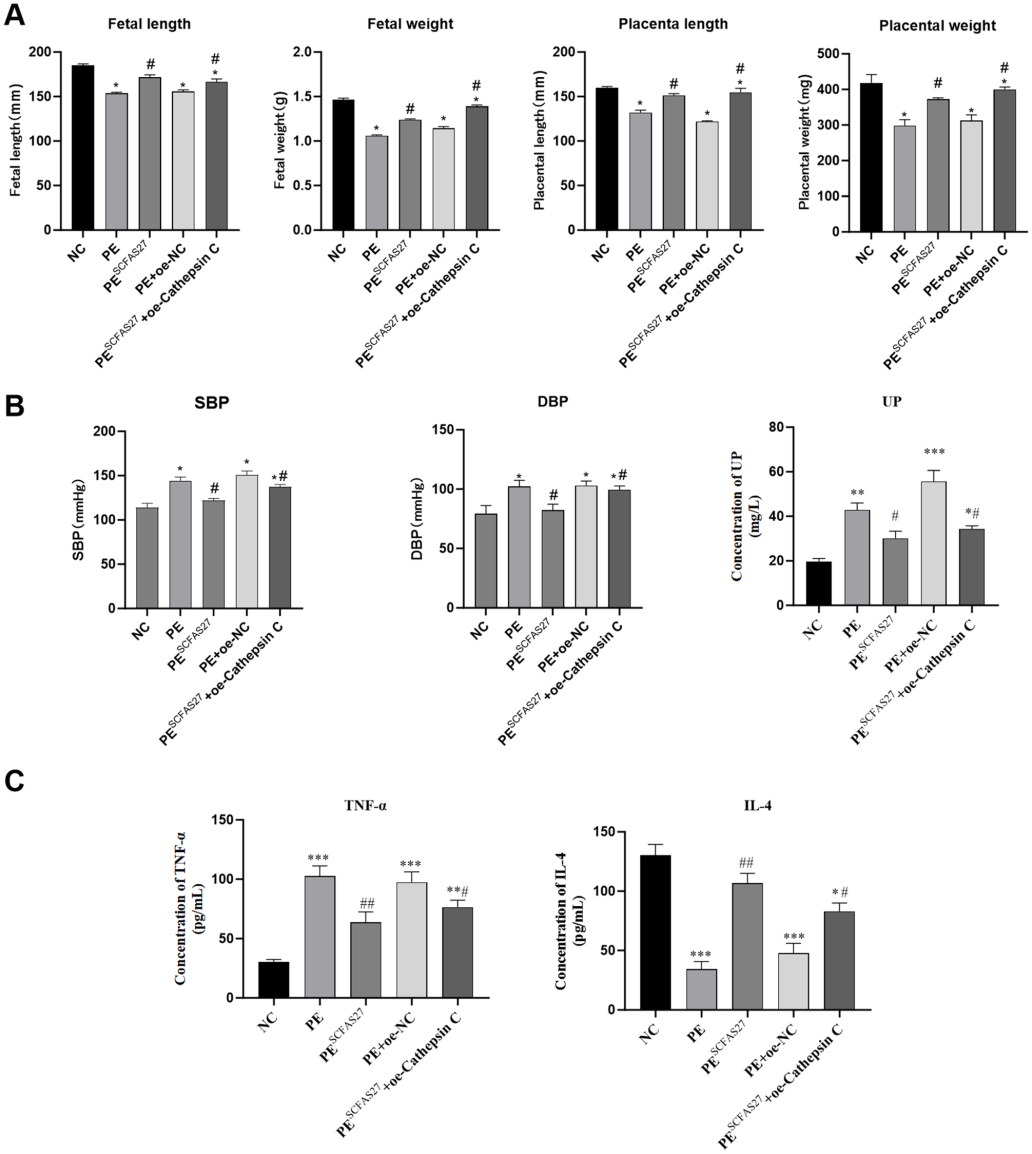
SCFAs 27 can significantly alleviate the pathological abnormalities in PE rats. SCFA supplementation alleviates core pathological features in a rat model of PE. **(A)** Placental weight, fetal weight, and fetal length, showing that SCFAs ameliorated PE-associated fetal growth restriction. **(B)** Longitudinal measurements of systolic blood pressure (SBP) and diastolic blood pressure (DBP) during gestation. SCFA treatment significantly attenuated L-NAME-induced hypertension. **(C)** Serum levels of TNF-α and IL-4. SCFAs reversed the pro-inflammatory cytokine profile. Data are presented as mean ± SD (n=9 rats per group). Statistical significance was determined by one-way ANOVA with Tukey’s *post-hoc* test. ^*^P < 0.05, ^**^P < 0.01, ^***^P < 0.001 vs. Control group. ^#^P < 0.05, ^##^P < 0.01 vs. LPS group.

**Figure 6:**
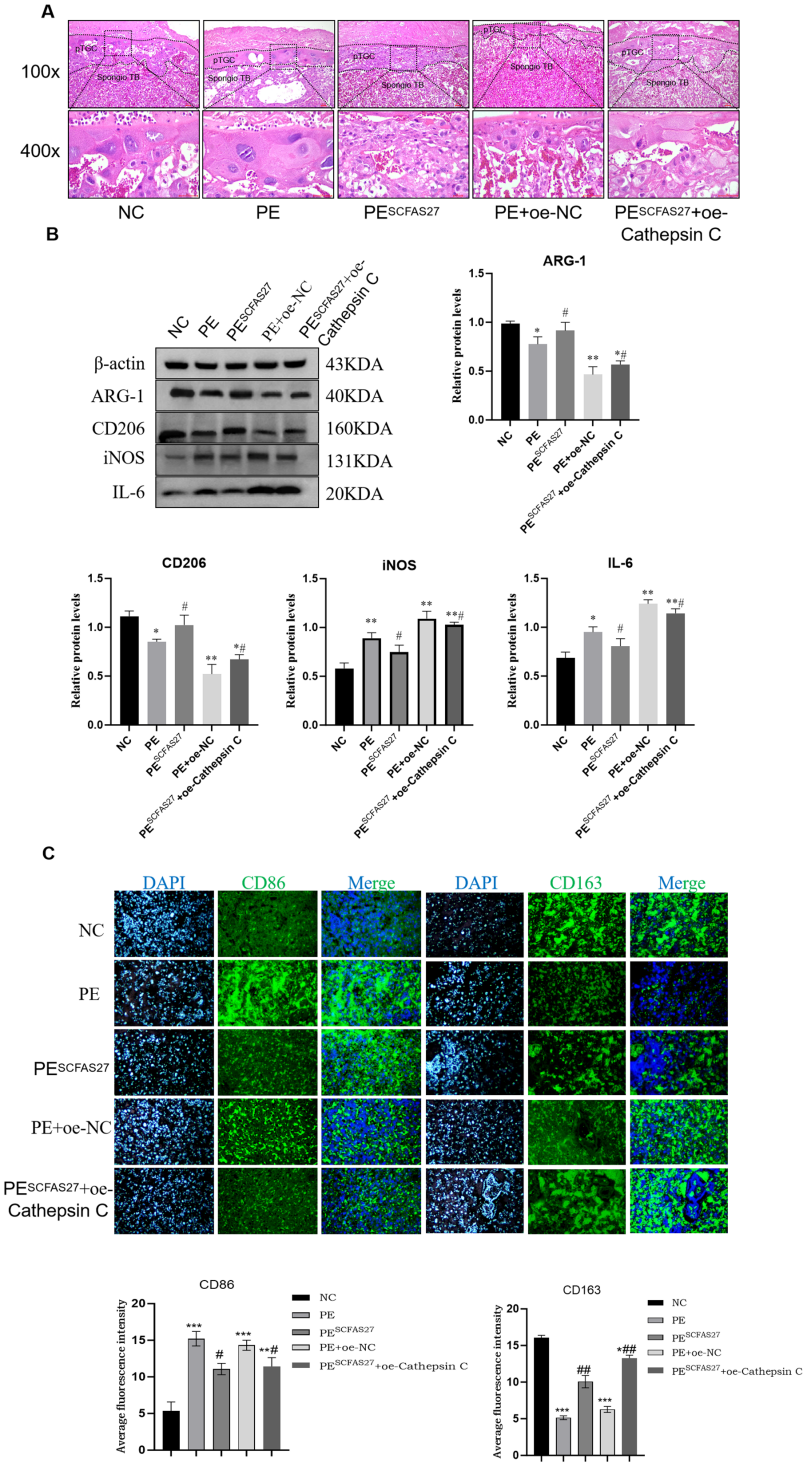
SCFAs27 intervention improves the phenotype of PE rats by inhibiting Cathepsin (C) **(A)** Representative H&E staining of placental sections. The PE group showed impaired spiral artery remodeling and infarction area (black box), which improved after SCFA treatment. Scale bars, 100 and 400 μm. **(B)** Western blot analysis of M1 (iNOS, IL-6) and M2 (CD206, Arg1) macrophage polarization markers in placental tissues. **(C)** Immunofluorescence staining and quantification of M1 marker CD86 and M2 marker CD163 in placental sections. Nuclei were counterstained with DAPI (blue). The M1/M2 ratio was significantly increased in the PE group and returned to normal after SCFA treatment. Scale bar, 20 μm. Data are presented as mean ± SD (n = 3 independent experiments). Statistical significance was determined by one-way ANOVA and Tukey’s *post hoc* test;. ^*^P < 0.05, ^**^P < 0.01, ^***^P < 0.001 vs. Control group. ^#^P < 0.05, ^##^P < 0.01 vs. PE group.

In comparison to the NC group, rats in the PE group exhibited standard pathological signs. Placental weight was significantly reduced (P < 0.05), as were fetal weight and body length (P < 0.05), indicating fetal growth restriction (FGR) (**Figure 5A**). Additionally, significant increases in systolic and diastolic blood pressure (by approximately 1.1-fold, P < 0.05) and elevated protein excretion in urine measured over 24 hours (P < 0.01) were observed (**Figure 5B**). ELISA results indicated pronounced systemic inflammation in the PE group, with significantly elevated serum TNF-α (P < 0.001) and reduced IL-4 (P < 0.01) (**Figure 5C**). Treatment with an SCFA mixture (PESCFAs27) markedly alleviated these pathological features. Placental and fetal development metrics were improved (P < 0.05, **Figure 5A**), blood pressure and proteinuria were significantly reduced (all P < 0.05, **Figure 5B**), and systemic inflammation was mitigated, as shown by reduced TNF-α and increased IL-4 levels (both P < 0.01, **Figure 5C**). Histological examination of placental tissue from the PE group revealed substantial impairment, such as insufficient trophoblast invasion and impaired spiral artery remodeling, narrowed vascular lumens, and focal infarctions, which were attenuated by SCFA treatment (**Figure 6A**). At the molecular level, Western blot analysis showed that the PE group exhibited upregulation of M1 macrophage markers (iNOS, P < 0.01; IL-6, P < 0.05) and downregulation of M2 markers (CD206 and Arg1, both P < 0.05) in placental tissue. Immunofluorescence staining confirmed an increased fraction of CD86^+^ (M1) macrophages and a decreased fraction of CD163^+^(M2) macrophages in the PE group, resulting in an elevated M1/M2 ratio (P < 0.01, compared to NC). SCFA intervention (PESCFAs27 group) promoted a significant shift toward M2 macrophage polarization, as indicated by a decreased fraction of CD86^+^cells, an increased fraction of CD163^+^cells, and a consequently reduced M1/M2 ratio (all P < 0.05, compared to PE, **Figures 6B, C**). To definitively test whether cathepsin C serves as the essential downstream mediator of SCFAs, we performed a rescue experiment by overexpressing cathepsin C during SCFA supplementation (PESCFAs27 +oe-Cathe psin C group). The rationale was that if SCFAs act primarily by suppressing cathepsin C, then forcing its expression should reverse the therapeutic benefits. Crucially, when cathepsin C was overexpressed in the context of SCFA treatment (PESCFAs27+ oe-Cathepsin C group), the beneficial effects of SCFAs on macrophage polarization were markedly abolished. The M1/M2 ratio in this group was significantly higher than that in the PESCFAs27 group (P < 0.01) and was statistically indistinguishable from that in the PE model group (P > 0.05). This demonstrates that cathepsin C overexpression fully reverses the SCFA-induced reprogramming of macrophages.

To assess the central role of cathepsin C in SCFA-mediated protection, we generated cathepsin C-overexpressing groups within the PE model. Compared to the empty vector control (PE+oe-NC), cathepsin C overexpression (PE+oe-Cathepsin C) worsened PE phenotypes and enhanced pro-inflammatory responses (P < 0.05), confirming its pathogenic role. Crucially, when cathepsin C was overexpressed during SCFA treatment (PESCFAs27+oe-Cathepsin C), the protective effects of SCFAs on maternal hypertension and proteinuria were significantly abrogated (**Figure 5B**). In contrast, the beneficial effects of SCFAs on fetal and placental growth parameters (fetal weight, fetal length, and placental weight) were not fully reversed by cathepsin C overexpression. This group exhibited higher blood pressure, increased proteinuria, elevated TNF-α, lower IL-4, sustained high cathepsin C expression, elevated levels of M1 markers and reduced levels of M2 markers.

In summary, this study demonstrates that SCFAs ameliorate core PE features by suppressing placental cathepsin C expression, with cathepsin C overexpression largely negating their benefits on maternal hypertension, proteinuria, and inflammation. It is notable, however, that the protective effects of SCFAs on fetal growth parameters were not fully reversed, thereby implying the involvement of additional, cathepsin C-independent mechanisms in safeguarding fetal development.

### 3.5 SCFAs regulate macrophage polarization via cathepsin C

**Figure 7:**
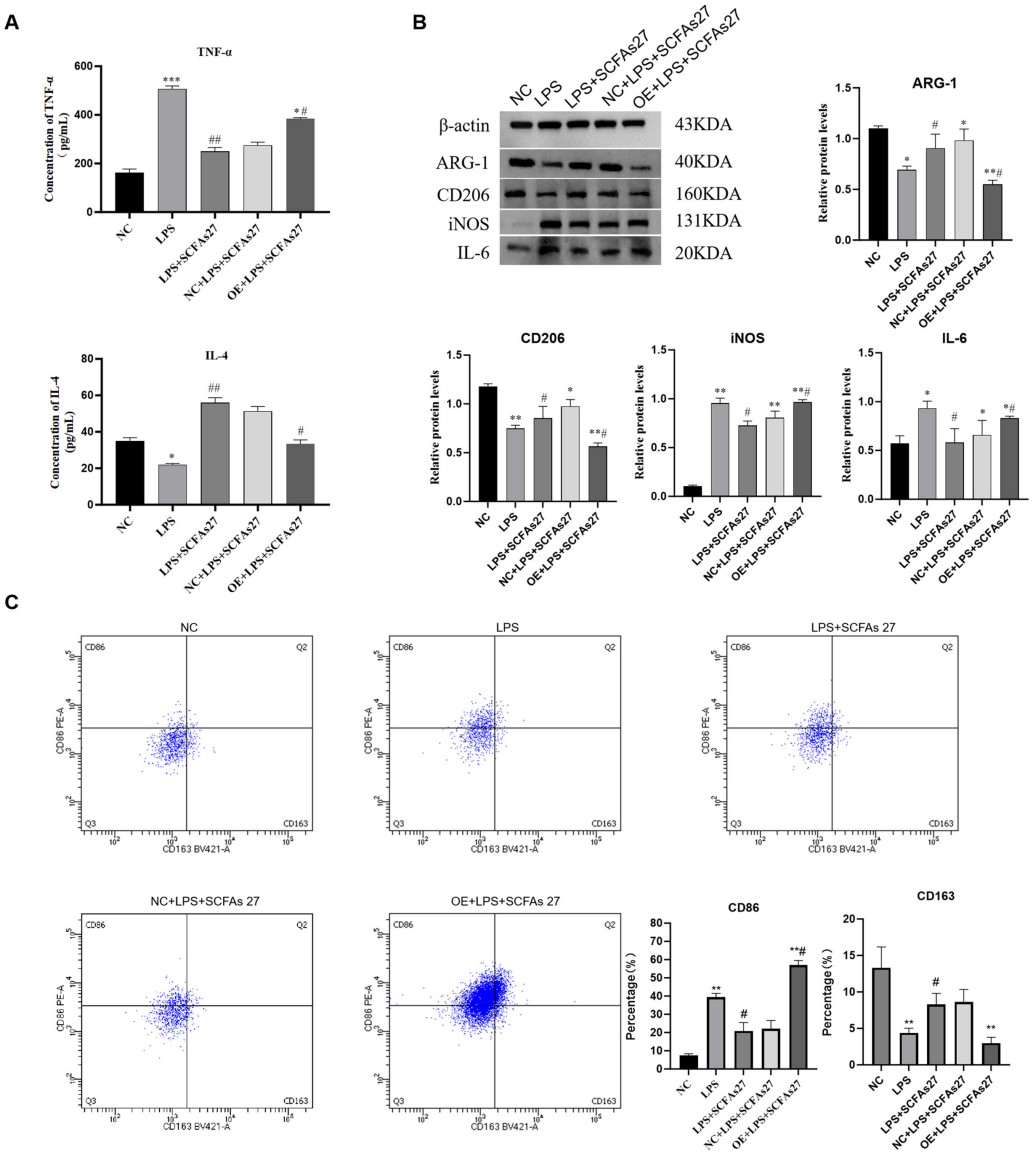
SCFAs regulate macrophage polarization through cathepsin C *in vitro*. RAW264.7 macrophages were stimulated with LPS to induce inflammation, with or without SCFA treatment and/or Cathepsin C overexpression (oe-Cathepsin C). **(A)** ELISA analysis of TNF-α and IL-4 levels in cell culture supernatants. **(B)** Western blot analysis of M1 (iNOS, IL-6) and M2 (CD206, Arg1) macrophage markers. **(C)** Flow cytometry analysis of surface markers CD86 (M1) and CD163 (M2) on CD11b+F4/80+ macrophages. The scatter plots (left) and quantitative bar graph (right) of the M1/M2 ratio are shown. SCFA treatment promoted M2 polarization, an effect that was abolished by Cathepsin C overexpression. Data are presented as mean ± SD (n=3 independent experiments). Statistical significance was determined by one-way ANOVA with Tukey’s *post-hoc* test. ^*^P < 0.05, ^**^P < 0.01, ^***^P < 0.001 vs. Control group. ^#^P < 0.05, ^##^P < 0.01, ^###^P < 0.001 vs. LPS group.

To investigate the molecular mechanisms through which SCFAs and cathepsin C modulate macrophage polarization in response to LPS-induced inflammation, we conducted a systematic series of interventions using the RAW264.7 cell model. Compared to the control group, LPS stimulation significantly increased TNF-α secretion and reduced IL-4 levels in the cell supernatant (both P < 0.01; **Figure 7A**). Flow cytometry indicated a pronounced shift toward the M1 phenotype in the LPS-treated group, with a larger proportion of CD86^+^ macrophages and a reduced fraction of CD163^+^ M2 macrophages, leading to a substantially elevated M1/M2 ratio (P < 0.01; **Figure 7C**). Western blot analysis further confirmed elevated expression of the pro-inflammatory markers iNOS and IL-6, alongside reduced levels of the M2 markers CD206 and Arg1, confirming the successful establishment of an inflammatory model (**Figure 7B**). Treatment with the SCFA mixture (SCFAs27) effectively mitigated these inflammatory responses: it significantly lowered TNF-α and increased IL-4 secretion (P < 0.05), reduced the M1/M2 ratio (P < 0.01), downregulated iNOS and IL-6, and enhanced expression of CD206 and Arg1 (P < 0.05), underscoring the strong anti-inflammatory and pro-M2 polarization effects of SCFAs *in vitro*. Western blot also showed that LPS stimulation markedly increased cathepsin C protein expression (P < 0.001), while SCFA treatment effectively suppressed this upregulation (P < 0.01 vs. LPS group), consistent with *in vivo* observations. Notably, overexpression of cathepsin C in the context of SCFA administration substantially diminished their protective influence, resulting in increased TNF-α, reduced IL-4 (P < 0.05), an elevated M1/M2 ratio (P < 0.05), and rebound expression of iNOS and IL-6, accompanied by decreased CD206 and Arg1 (P < 0.05). These results provide direct evidence that SCFAs promote M2 macrophage polarization primarily through suppression of cathepsin C. Cathepsin C overexpression counteracts SCFA-induced anti-inflammatory effects, favoring a pro-inflammatory M1 state. Thus, this study molecularly establishes the core role of the SCFA–Cathepsin C–Macrophage Polarization axis in regulating inflammatory responses.

## 4 Discussion

Our study elucidates a novel immunological pathway in preeclampsia (PE) pathogenesis, centering on the “gut microbiota–SCFAs–cathepsin C–macrophage polarization” axis. We first established a clinical link between gut dysbiosis, SCFA deficiency, and upregulated placental cathepsin C in PE patients. Subsequently, through interventional studies in a rat model, we demonstrated that SCFA supplementation alleviates disease phenotypes and promotes protective M2 macrophage polarization by suppressing cathepsin C. Crucially, the reversal of SCFA benefits upon cathepsin C overexpression definitively establishes it as a key downstream effector. These integrated findings position cathepsin C as a critical molecular bridge connecting gut microbiota-derived metabolites to disrupted placental immune tolerance.

The core pathological features of preeclampsia (PE) include defective remodeling of placental spiral arteries and dysregulation of immune responses at the maternal-fetal interface. Emerging evidence has identified the gut microbiota as a pivotal environmental factor influencing PE development, chiefly through its role in modulating systemic and placental immunity via the “gut–placenta axis.” Clinical metagenomic studies have revealed substantial gut microbial dysbiosis in individuals with PE, characterized by a greater than 50% decrease in SCFA-producing bacterial populations and a 2.1-fold increase in pro-inflammatory genera. These shifts correlate with suppressed activity in SCFA biosynthetic pathways, ultimately reducing the inhibitory influence of SCFAs on histone deacetylases (HDACs) (22).SCFAs function as essential mediators in host-microbiota crosstalk and help maintain immune homeostasis through two principal mechanisms: activation of G protein-coupled receptors (GPCRs), which stimulates the release of the anti-inflammatory cytokine IL-10, and inhibition of HDAC activity, which attenuates NF-κB and p38 MAPK signaling and encourages macrophages to shift to the M2 phenotype (23, 24). Nevertheless, the precise molecular targets through which SCFAs influence placental macrophage polarization remain inadequately defined. Cathepsin C, a lysosomal protease frequently overexpressed in inflammatory conditions, promotes M1 macrophage polarization by activating the NLRP3/NF-κB pathway (25, 26). However, its specific involvement in PE pathogenesis and its potential linkage to the gut microbiota–SCFA axis have yet to be systematically explored.

Prior investigations have revealed major imbalances in the gut microbial community among those with preeclampsia (PE), marked by a pronounced decrease in SCFA-producing bacteria—including Blautia, Collinsella, and Akkermansia muciniphila—alongside an expansion of pro-inflammatory genera such as Parabacteroides and Odoribacter. This structural shift, further supported by KEGG functional profiling, correlates with the suppression of essential SCFA synthesis pathways and a consequent decline in SCFA production capacity (27, 28).By combining clinical 16S rRNA sequencing with placental molecular profiling, our study revealed substantial upregulation of cathepsin C in placental tissues from PE patients compared to controls. Importantly, a negative correlation was observed between cathepsin C expression and SCFA-producing gut bacteria, whereas a positive correlation was found with systolic blood pressure and 24-hour urinary protein excretion. This positions cathepsin C, for the first time, as a key molecular node linking gut microbiota-derived SCFA deficiency to placental dysfunction in PE. Existing literature also documents aberrant expression of other cathepsins—such as cathepsin B and L—in PE placentas, where their levels correlate with clinical severity (29, 30). As a lysosomal cysteine protease, cathepsin C is known to promote trophoblast apoptosis and disrupt spiral artery remodeling through mechanisms involving inflammasome activation and extracellular matrix degradation. Our study delineates a novel pathway wherein SCFAs ameliorate PE by targeting cathepsin C. Beyond their known roles in GPCR and HDAC signaling, our rescue experiment positions cathepsin C suppression as a pivotal mechanism for SCFA action in placental immunity. To delineate the molecular pathway downstream of cathepsin C, we integrated our data with established protease biology. Our findings support a model wherein cathepsin C acts as a pro-inflammatory amplifier, critically positioned to sustain M1 macrophage polarization. Mechanistically, cathepsin C, a master activator of several granule-associated serine proteases, is poised to cleave and activate protease-activated receptors (PARs) and enhance the processing of key inflammatory mediators. This activity perpetuates the activation of the NF-κB and p38 MAPK signaling pathways, which are central transcriptional drivers of classic M1 markers, including TNF-α, IL-6, and iNOS. Concurrently, cathepsin C facilitates the priming and full activation of the NLRP3 inflammasome, leading to the caspase-1-dependent maturation and secretion of IL-1β, another potent M1-polarizing cytokine. Consequently, the SCFA-driven downregulation of placental cathepsin C disrupts this pro-inflammatory circuit. By attenuating cathepsin C expression, SCFAs reduce signaling through NF-κB/p38 MAPK and limit NLRP3 inflammasome activity. This dual suppression shifts the cytokine balance away from M1-driving signals, thereby relieving the inherent suppression on M2 transcriptional programs. This altered milieu permits the upregulation of M2-associated markers such as Arg-1, IL-10, and CD206, effectively promoting the resolution of inflammation and the re-establishment of immune tolerance at the maternal-fetal interface. Thus, cathepsin C emerges not as a passive biomarker but as an active regulatory node, whose suppression by SCFAs is instrumental in redirecting macrophage polarization towards a protective phenotype (31, 32). Interventional studies in animals confirmed that SCFA administration downregulated placental cathepsin C, concurrently alleviating hypertension and placental damage. In contrast, overexpression of cathepsin abolished the beneficial effects of SCFAs, solidifying its role as a specific downstream effector in SCFA-mediated protection. This understanding creates new opportunities for therapeutic approaches in PE. Future research efforts aim to clarify the individual contributions of different SCFAs to cathepsin C regulation and determine their spatiotemporal roles in guiding placental macrophage polarization.

The origin and fate of the gut microbiota dysbiosis observed in PE patients warrant further discussion. This divergence likely stems not from a single factor but from a combination of pre-pregnancy microbiota baseline, dietary habits during gestation, host genetics, and the systemic inflammation and metabolic disturbances inherent to PE itself (33, 34). Notably, the pathological state of PE (hypertension, proteinuria, and systemic inflammation) may exacerbate dysbiosis by altering gut permeability and the local microenvironment, creating a vicious cycle. Regarding the timing, evidence suggests that certain microbial signatures may exist early in pregnancy or even pre-conception and worsen as gestation progresses (35). As for postpartum recovery, while some studies indicate a trend toward normalization after delivery, women with a history of PE may retain some aberrant microbial and metabolic features long-term, potentially linked to their elevated future cardiovascular risk (36, 37). Thus, the PE-associated microbiota dysbiosis is likely both a cause and a consequence of the disease, with potential lasting effects.

One finding from our animal experiments warrants further consideration. The protective effects of SCFAs on maternal hypertension and proteinuria were largely abolished by Cathepsin C overexpression. However, the positive influence of SCFAs on fetal and placental growth was not fully reversed. This outcome suggests that the mechanisms governing maternal symptoms and fetal development may be partially distinct. We suspect this could be because the SCFA intervention, administered earlier, had already induced some lasting changes in placental structures such as vascular remodeling. Once these structural foundations were established, they proved somewhat resilient to being completely overturned by the subsequent single manipulation of Cathepsin C overexpression. In this light, the primary function of Cathepsin C appears to be regulating maternal immune and vascular homeostasis, with a more limited capacity to influence an established fetal growth program. This unexpected discrepancy, therefore, helps us more precisely define the principal role of Cathepsin C in the pathological process of preeclampsia.

Given the recognized function of SCFAs in enhancing maternal–fetal immune tolerance by downregulating cathepsin C, we performed additional interventions to confirm the pivotal role of cathepsin C in the SCFA-mediated placental defense pathway. In this investigation, administration of exogenous SCFAs significantly downregulated placental cathepsin C levels while concurrently alleviating core PE pathological manifestations. In contrast, forced overexpression of cathepsin C completely negated the protective benefits of SCFA supplementation, reverting hypertension and proteinuria to pathological levels. This bidirectional approach—combining gain- and loss-of-function strategies—definitively establishes cathepsin C as the critical molecular mediator through which SCFAs exert placental protection. Furthermore, this regulatory mechanism exhibits notable specificity. Elevated cathepsin C expression within placental macrophages enables precise targeting of key immune subsets at the maternal-fetal interface. Significantly, SCFAs modulate macrophage polarization through cathepsin C inhibition via a mechanism independent of the conventional HDAC-mediated epigenetic pathway, distinguishing it from the broader anti-inflammatory effects associated with the SCFA–GPCR/HDAC axis (38, 39). Furthermore, existing research underscores the importance of protease activity in placental pathology. For example, Ulrich et al. demonstrated that matrix metalloproteinase 9 (MMP9) degrades extracellular matrix components such as collagen and laminin, markedly enhancing uterine contractility and suggesting a potential role in aberrant placental vascular remodeling (40). In alignment with this, our results indicate that cathepsin C overexpression directly undermines placental barrier integrity, highlighting another protease-mediated pathway contributing to PE pathogenesis.

This study provides the first comprehensive delineation of the molecular mechanism by which SCFAs specifically suppress cathepsin C expression to promote M2 macrophage polarization and reestablish immune tolerance at the maternal-fetal interface. These findings not only extend beyond the classical SCFA–GPCR/HDAC paradigm of generalized immunomodulation but also define a central role for the “gut microbiota–SCFA–cathepsin C–macrophage polarization” axis in PE pathogenesis. By integrating evidence from clinical cohorts and animal models, we propose cathepsin C-targeted intervention as a novel strategic framework for PE prevention and treatment. Future studies should prioritize investigating the crosstalk between this axis and pathways controlling placental vascular remodeling and pyroptosis, to further decode the integrated mechanisms underlying immune dysregulation within the maternal-fetal microenvironment.

The promising therapeutic effects of SCFAs in our experimental models warrant a considered discussion on their clinical translation for preeclampsia. The most feasible and immediately applicable strategy likely involves dietary interventions, such as high-fiber or prebiotic supplementation, to naturally boost endogenous SCFA production, an approach that aligns with general prenatal nutrition guidelines and presents a favorable safety profile. Future clinical trials are needed to establish the efficacy of such regimens and to define the optimal timing, perhaps targeting high-risk women early in gestation. Critically, long-term follow-up studies will be essential to determine if correcting SCFA deficiency can mitigate not only the acute symptoms of preeclampsia but also the associated lifelong cardiovascular risks for both mother and child. Our findings, which link specific microbial metabolites to a defined molecular pathway, further suggest a path for precision medicine, where at-risk individuals could be identified by their gut microbiome and metabolic profile for targeted prophylactic therapy.

This study has several limitations that should be considered when interpreting the results. Firstly, as noted in our methods, the clinical cohort was recruited from a single center and the sample size, while sufficient to identify robust group differences, was not large enough to permit comprehensive adjustment for potential confounding factors such as maternal age, pre-pregnancy BMI, parity, dietary habits, or genetic background. While our patient inclusion criteria were designed to create homogeneous groups, we acknowledge that the influence of these variables on the gut microbiota-SCFA-cathepsin C axis remains an open and important question. Secondly, we acknowledge the limitation of using the L-NAME-induced rat model and the RAW264.7 macrophage line. Our decision was grounded in the model’s robustness for studying the hypertensive and inflammatory endpoints central to our hypothesis and the cell line’s tractability for dissecting signaling pathways. However, the L-NAME model primarily reflects the maternal systemic response and endothelial dysfunction, and may not fully capture the initial placental triggers of some PE subtypes. Furthermore, the translation of animal-derived SCFA doses to clinically relevant human equivalents remains a complex challenge. While the dosage used here was effective in rescuing PE phenotypes in our model, a systematic dose-response relationship was not established. Future research is warranted to determine the optimal therapeutic window and to explore safe delivery strategies for human pregnancy.

## Data Availability

The data presented in the study are deposited in the NCBI Sequence Read Archive (SRA) under the BioProject repository,accession number PRJNA1357918.

## Supplementary material

Supplementary Figure 1 Verification of animal experiment transfection results (QPCR results).

Supplementary Figure 2 Verification of cell experiment transfection results (QPCR results).

Supplementary Figure 3 Rat transfection.

Supplementary Figure 4 Rat fetus.

Supplementary Figure 5 Rat placenta.

Supplementary Figure 6 Animal experiment protein.

Supplementary Figure 7 Cell experimental protein.

Supplementary Figure 8 Comprehensive functional profiling details (**Figure 3**).
