# Supplementary figures and images for: Unraveling the gut microbiota–SCFAs–cathepsin C pathway in preeclampsia: a novel therapeutic target
Source: Front Immunol. 2025 Nov 17;16:1700781. doi: 10.3389/fimmu.2025.1700781 (PMC12665775; doi:10.3389/fimmu.2025.1700781)

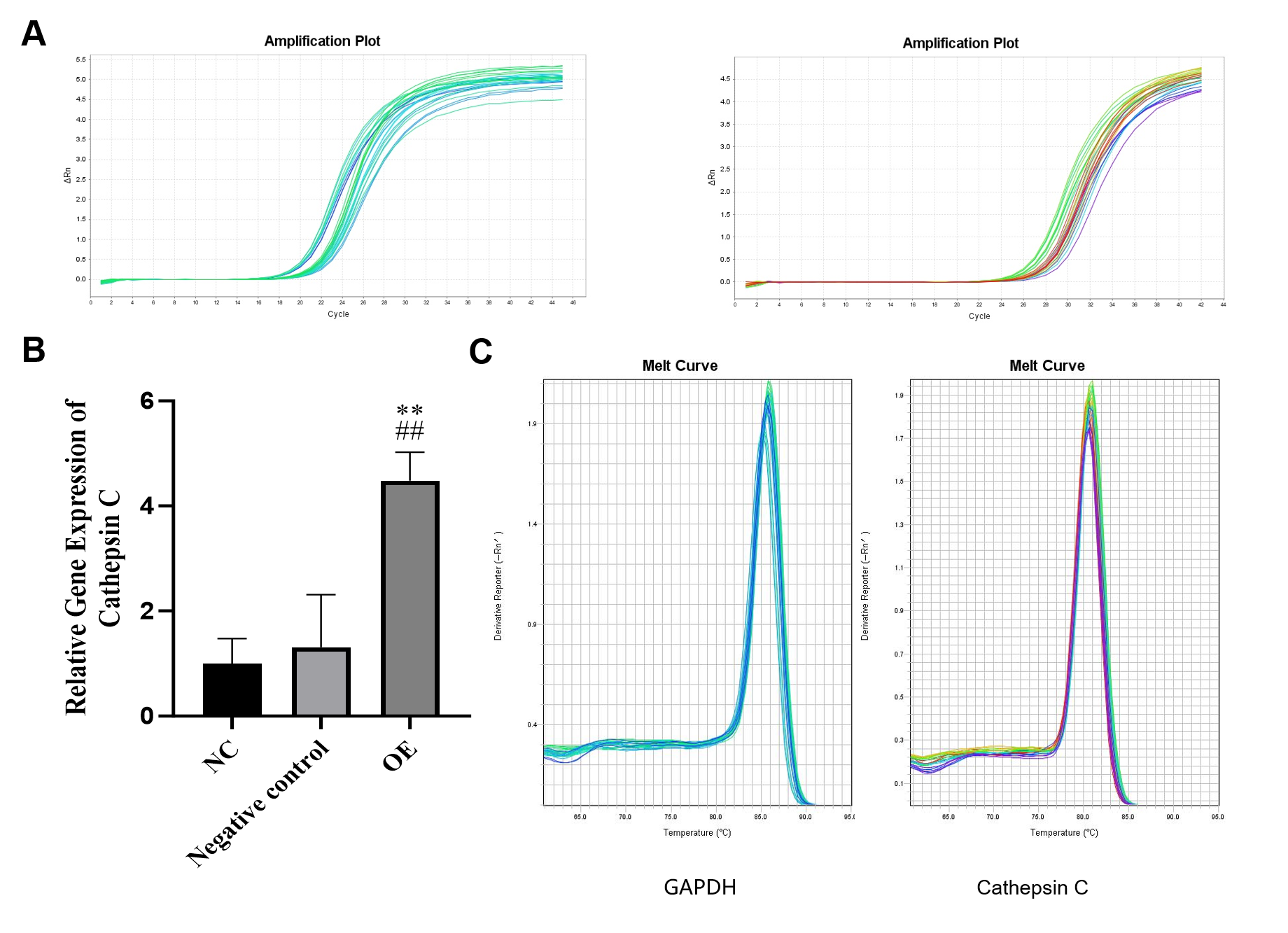

Supplement: Supplementary Figure 1 — Verification of animal experiment transfection results (QPCR results). [file Image1.tif]

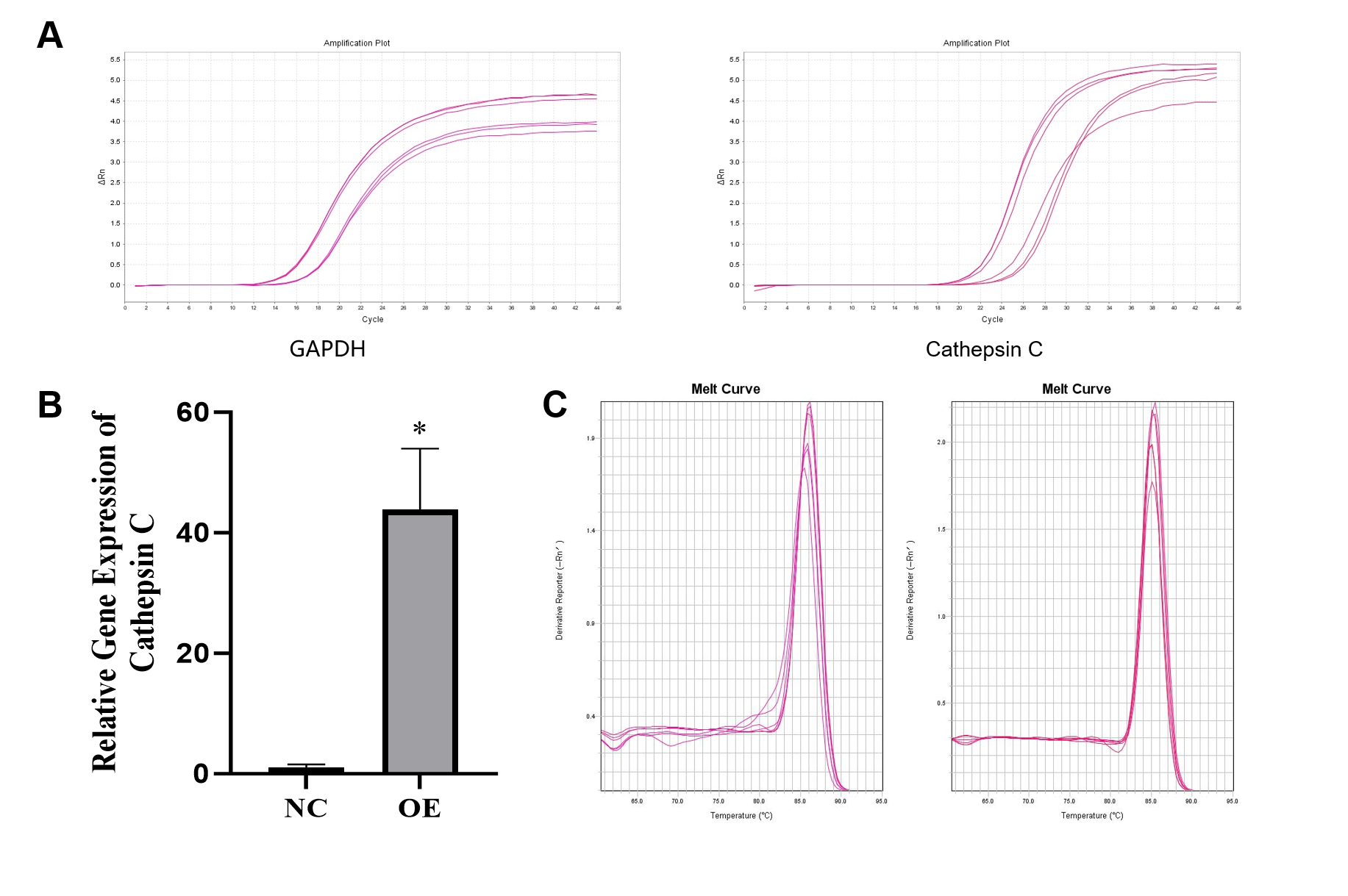

Supplement: Supplementary Figure 2 — Verification of cell experiment transfection results (QPCR results). [file Image2.tif]

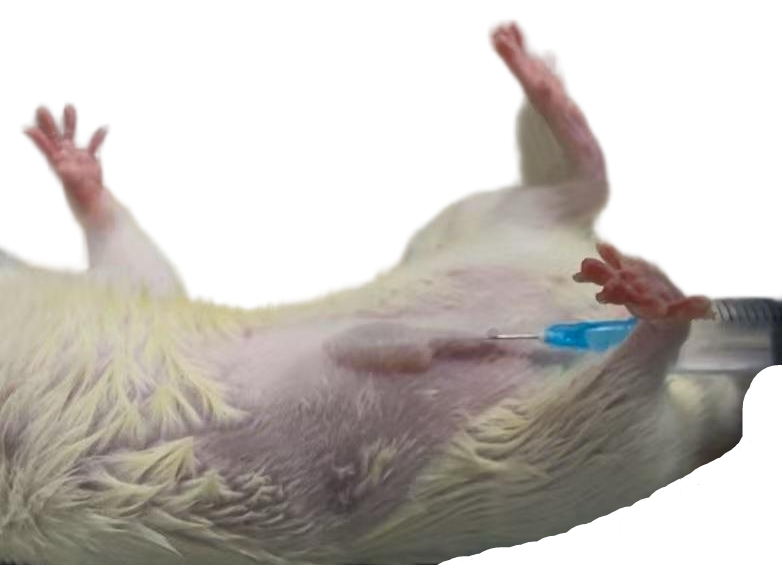

Supplement: Supplementary Figure 3 — Rat transfection. [file Image3.tif]

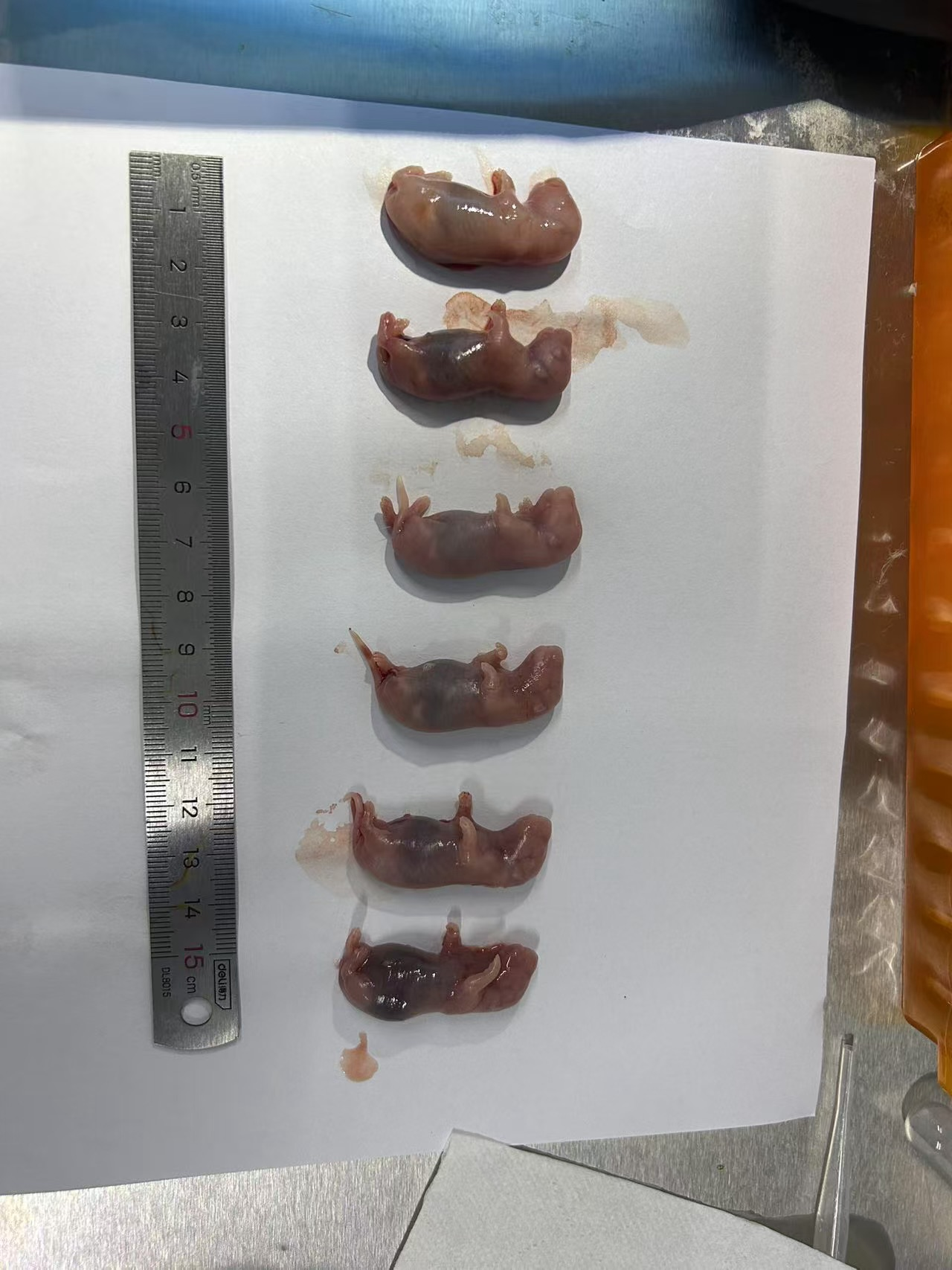

Supplement: Supplementary Figure 4 — Rat fetus. [file Image4.tif]

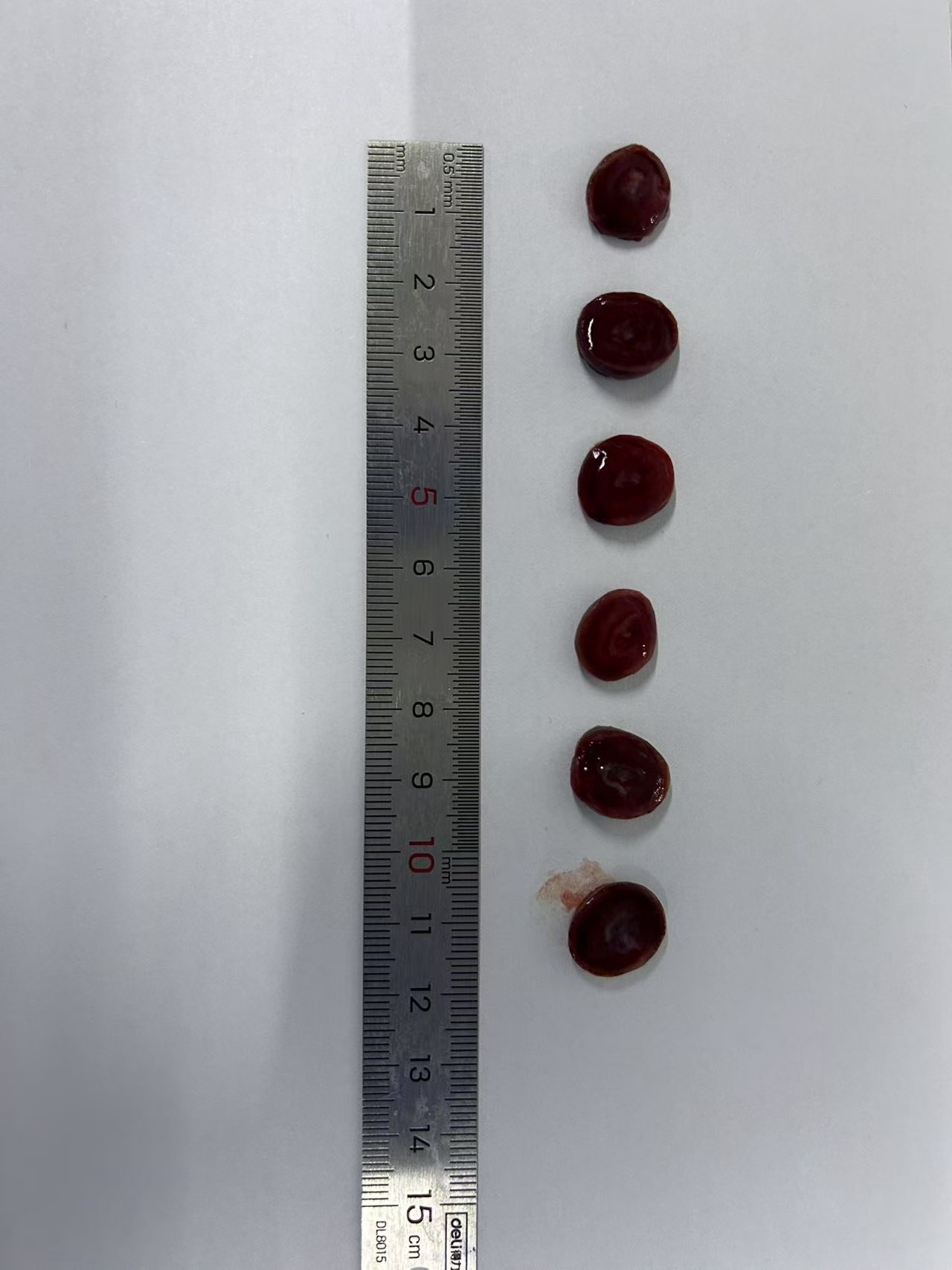

Supplement: Supplementary Figure 5 — Rat placenta. [file Image5.tif]

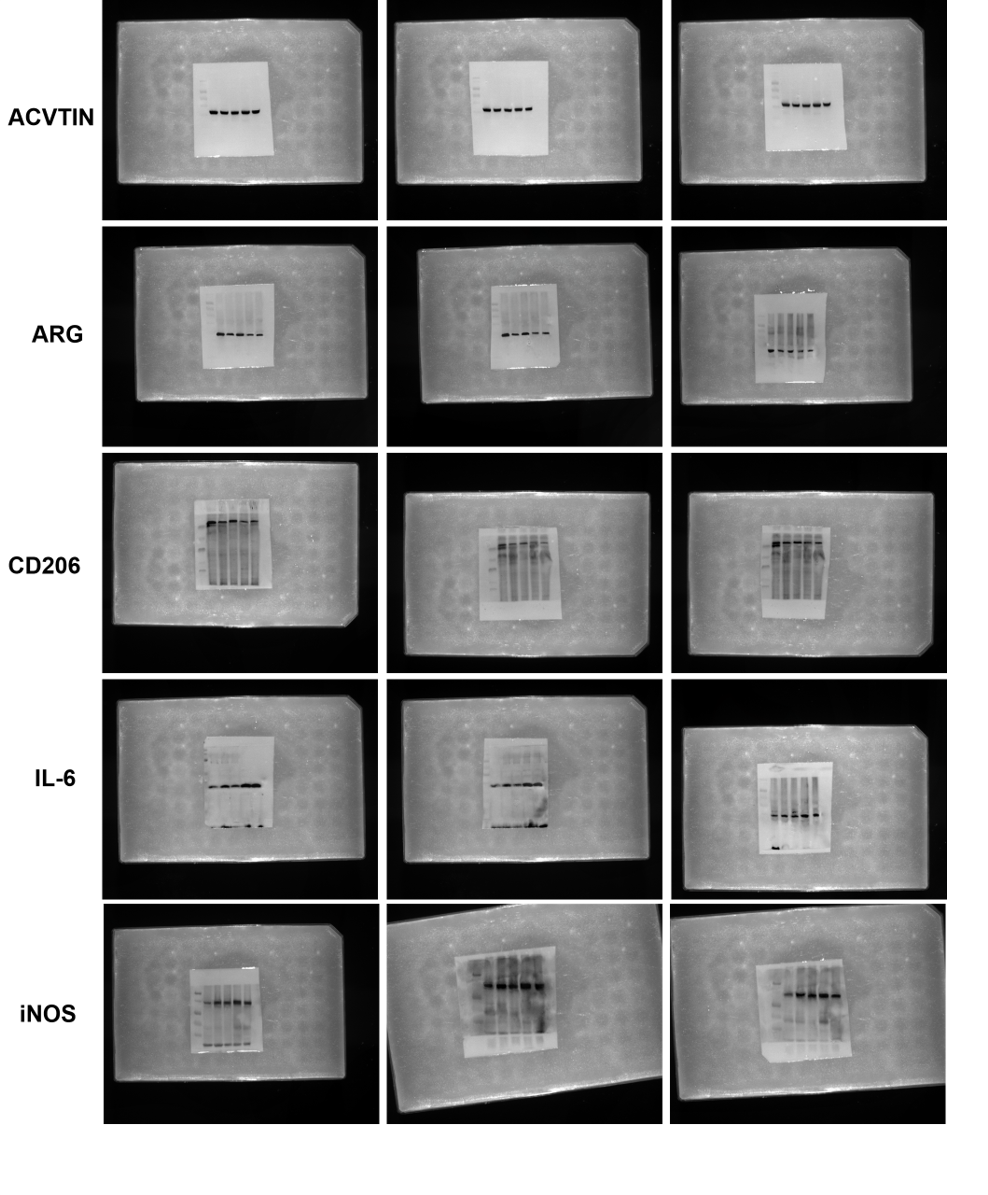

Supplement: Supplementary Figure 6 — Animal experiment protein. [file Image6.tif]

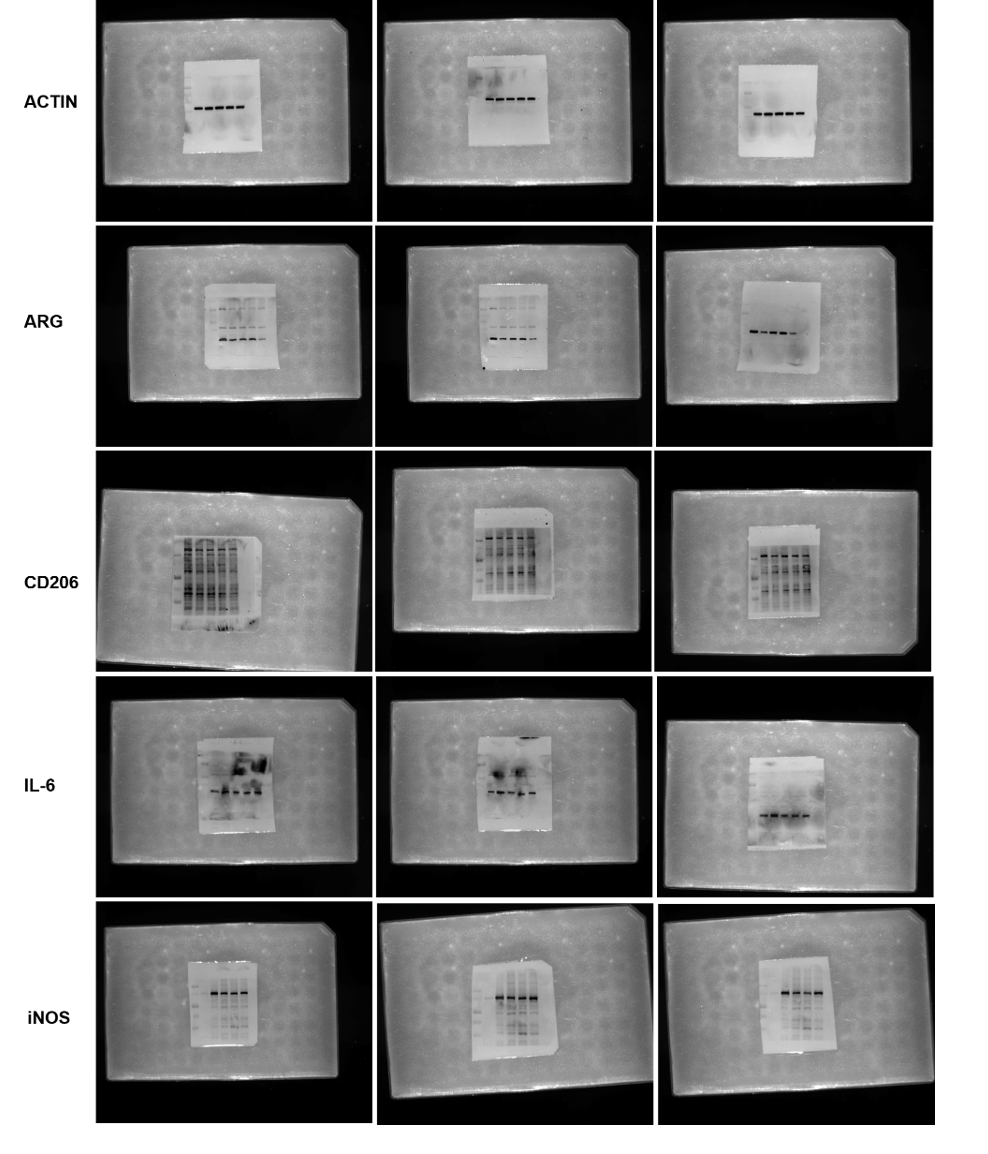

Supplement: Supplementary Figure 7 — Cell experimental protein. [file Image7.tif]
