# Supplementary material for: Unraveling the gut microbiota–SCFAs–cathepsin C pathway in preeclampsia: a novel therapeutic target
Source: Front Immunol. 2025 Nov 17;16:1700781. doi: 10.3389/fimmu.2025.1700781 (PMC12665775; doi:10.3389/fimmu.2025.1700781)
Supplement: Supplementary file 9 [file Table1.docx]

**Supplementary Table 1: Antibodies used in this study**

| **Antibody Target** | **Application** | **Host Species & Clonality** | **Vendor** | **Catalog Number** | **Dilution Used** |
| --- | --- | --- | --- | --- | --- |
| **Cathepsin C** | WB, IF | Rabbit Monoclonal | Wuhan Sanying Biotechnology | 30790-1-AP | 1:1000 (WB) 1:200 (IF) |
| **CD86** | Flow Cytometry, IF | Rat Monoclonal | BioLegend | 105012 | 1:100 (Flow) 1:200 (IF) |
| **CD163** | Flow Cytometry, IF | Rat Monoclonal | Santa Cruz | sc-58965 | 1:50 (Flow) 1:200 (IF) |
| **iNOS** | WB | Rat Monoclonal | Hangzhou Huidan Biotechnology | A96190 | 1:1000 |
| **IL-6** | WB | Rat Monoclonal | Hangzhou Huidan Biotechnology | A13865 | 1:1000 |
| **CD206** | WB | Rat Monoclonal | Beijing Biosynthesis Biotechnology | bs-4727R | 1:1000 |
| **Arg1** | WB | Rat Monoclonal | Hangzhou Huidan Biotechnology | A78858 | 1:1000 |
| **β-actin** | WB | Rabbit Polyclonal | Beijing Biosynthesis Biotechnology | bs-0061R | 1:5000 |
| **HRP-conjugated Goat Anti-Rabbit IgG** | WB | Goat | Beyotime Biotechnology | A0208 | 1:5000 |
